# Retraction Note: Tobacco-smoking-related prevalence of methanogens in the oral fluid microbiota

**DOI:** 10.1038/s41598-025-88912-6

**Published:** 2025-02-11

**Authors:** Ghiles Grine, Elodie Terrer, Mahmoud Abdelwadoud Boualam, Gérard Aboudharam, Hervé Chaudet, Raymond Ruimy, Michel Drancourt

**Affiliations:** 1 Aix Marseille Université, MEPHI, IRD, IHU Méditerranée Infection, Marseille, France; 2 Pôle Odontologie, Assistance Publique – Hôpitaux de Marseille, Marseille, France; 3 Faculté d’odontologie, Université d’Aix Marseille, Marseille, France; 4 Laboratoire de Bactériologie, Centre Hospitalier Universitaire de Nice, Hôpital de L’Archet II, Université Côte d’Azur, INSERM U1065, C3M, Team 6, Nice, France

Retraction of: *Scientific Reports* 10.1038/s41598-018-27372-7, published online 15 June 2018

Editors have retracted this Article.

After publication of this Article concerns about ethical oversight of this study were brought to the attention of the Editors. The Authors were not able to provide documentation of approval from an appropriate ethics committee for the work reported.

Ghiles Grine, Elodie Terrer, Mahmoud Abdelwadoud Boualam, Gérard Aboudharam, Raymond Ruimy, and Michel Drancourt did not respond to the correspondence from Editors about the retraction. Editors were not able to obtain the current contact details for Hervé Chaudet.

